# Aqueous Phase Synthesis of CuIn Alloy Nanoparticles and Their Application for a CIS (CuInSe_2_)-Based Printable Solar Battery

**DOI:** 10.3390/nano8040221

**Published:** 2018-04-06

**Authors:** Hideyuki Takahashi, Hironari Fujiki, Shun Yokoyama, Takayuki Kai, Kazuyuki Tohji

**Affiliations:** 1 Graduate school of Environmental studies, Environmentally Benign Systems, Tohoku University, Sendai 980-8579, Japan; hideyuki@mail.kankyo.tohoku.ac.jp (H.F.); shun.yokoyama.c2@tohoku.ac.jp (S.Y.); kazuyuki.tohji.a8@tohoku.ac.jp (K.T.); 2 Panasonic Corp. Manufacturing Technology and Engineering Div., Osaka 571-8502, Japan; kai.takayuki@jp.panasonic.com

**Keywords:** metal complex, control, aqueous phase synthesis

## Abstract

To apply CuInSe_2_ (CIS)-based printable solar batteries; an aqueous phase synthesis method of Cu-In (CI) alloy nanoparticles is studied. Metal complexes in the original solution are restricted to homogenized species by utilizing calculations. For example; [(Cu^2+^)(ASP^2−^)_2_] [ASP: the “body (C_4_H_5_O_4_N)” of aspartic acid (C_4_H_7_O_4_N)] is predominant in the pH 6–13 region (C_ASP_/C_Cu_ > 6); while In complexes can be restricted to [(In^3+^)(OH^−^)(EDTA^4−^)] (pH 10–12; C_EDTA_/C_In_ = 2) and/or [(In^3+^)(ASP^2−^)_2_] (pH 7–9; C_ASP_/C_In_ = 5). These results indicate that the added amount of complex reagents should be determined by calculations and not the stoichiometric ratio. The reduction potential of homogenized metal complex is measured by cyclic voltammetry (CV) measurements and evaluated by Nernst’s equation using the overall stability constants. CuIn alloy nanoparticles with a small amount of byproduct (In nanoparticles) are successfully synthesized. The CI precursor films are spin-coated onto the substrate using a 2-propanol dispersion. Then the films are converted into CIS solar cells; which show a maximum conversion efficiency of 2.30%. The relationship between the open circuit potential; short circuit current density; and fill factor indicate that smoothing of the CIS films and improving the crystallinity and thickness increase the solar cell conversion efficiency.

## 1. Introduction

Actualization of environmental problems has increased research that utilizes natural energy [1,2,3]. Recently, Si-based solar batteries have been widely used in effective natural energy conversion devices. However, the minimum energy payback time is ca. 1.5 years because the reduction of SiO_2_ to Si has an extremely large energy consumption (−856.7 J/mol) [4,5]. However, increasing the conversion efficiency will decrease the energy payback time, but drastically changing a Si-based solar battery’s efficiency is challenging as its actual efficiency has already reached 25% and the theoretical value is ca. 28% [6,7]. Therefore, various types of solar batteries have been vigorously researched. 

Thin film-based solar batteries [8] have been intensively studied due to their attractive characteristics, such as a relatively high efficiency [7], resource conservation [9,10], simple synthesis, etc. [11,12]. Since CuInSe_2_ (CIS)-based solar batteries are attractive solar cell materials [13] with a maximum efficiency up to 20.9% and long lifetimes in large scale modules, commercialization of CIS-based solar batteries is stimulated [14]. 

A CIS-based solar battery is mainly synthesized utilizing the gas phase deposition method [15,16]. This synthesis consumes a large amount of natural resources and has a high energy loss because the vapors of individual elements are widely spread and accumulate on the substrate in various concentrations [16,17,18]. Furthermore, the solar cell’s shape and size are limited by the reaction apparatus’ dimensions. Thus, many studies have tried to synthesize CIS materials by the chemical reduction method, which is an ecological and economical technique. For example, Ye et al., synthesized Cu-In alloy particles via chemical reduction in an organic solvent in which the Cu-In alloy precursor films were transformed into CuInSe_2_ films by a selenide procedure [19]. The resulting cells demonstrated a maximum energy conversion efficiency of 3.92% [19]. However, the total cost of this method is not low since this procedure requires an organic solvent, such as tetraethylene glycol (C_8_H_18_O_5_, TEG). Therefore, Shih-Hsien et al. synthesized Cu-In alloy nanoparticles in an aqueous phase using metal salts and triethylamine as a stabilizing reagent; selenide materials showed an energy conversion efficiency of 1.47% [20]. Unfortunately, the synthetic mechanism in the aqueous solution remains unclear, and their process cannot be widely applied to prepare other compositions or materials. 

Although metallic nanoparticles are easily synthesized in the liquid phase by the reduction of metal ions and/or complexes, core-shell materials and/or amorphous-like materials are sometimes formed. The former appears when the reduction rate of each metal ion and/or complex differs, while the latter is synthesized when a strong reducing agent is used. Therefore, to synthesize uniform and well-crystallized alloy nanoparticles, Takahashi et al. controlled the homogenization of the metallic complexes in an aqueous solution at room temperature by using calculations, EXAFS (extended X-ray absorption fine structure), and ESI-TOF-MS (electrospray ionization time of flight mass spectroscopy) analyses [21,22]. Consequently, uniform and well-crystallized alloy nanoparticles, such as Pd_20_Te_7_ and Bi_2_Te_3_, were successfully synthesized [22,23].

Co-deposition of Cu and In ions is relatively difficult since the reduction potentials of these ions differ greatly (0.342 V for Cu and −0.338 V for In) [24,25]. However, the reduction potential of the metal complex is not the same as that of the ion. This means that the difference in the reduction potential of each metal can be freely controlled using metal complex species (i.e., critical stability constants) and that of the ratio in aqueous solution should realize uniform and well-crystallized CuIn alloy nanoparticles. 

In this study, the synthesis and its mechanism to form Cu-In alloy nanoparticles are elucidated. Moreover, the synthesized materials are applied to form CIS-based printable solar batteries. 

## 2. Experimental

### 2.1. Calculations

Copper nitrate (Wako Chemicals, Japan, Cu(NO_3_)_2_•3H_2_O) and indium nitrate (Wako Chemicals, InNO_3_•3H_2_O) were used as the Cu and In precursor salts, respectively. Citric acid (Wako Chemicals, C_6_H_8_O_7_), maleic acid (Wako Chemicals, C_4_H_4_O_4_), glycine (Wako Chemicals, C_2_H_5_O_2_N), ethylenediamine (Wako Chemicals, C_2_H_8_N_2_, called as “EDA”), nitrilotriacetic acid (Wako Chemicals, C_6_H_9_O_6_N), ethylenediaminetetraacetic acid (Wako Chemicals, C_10_H_16_O_8_N_2_, “EDTA”), oxalic acid (Wako Chemicals, C_2_H_2_O_4_), and aspartic acid (Wako Chemicals, C_4_H_7_O_4_N) were used as complex reagents. The concentrations of Cu and/or In complexes at various pH values of the aqueous solution were calculated using the critical stability constants. The calculation method is described in the literature [21]. 

For example, briefly, calculation methods for Cu ion-aspartic acid-OH^−^-NO_3_^−^ system were performed as follows: Aspartic acid (C_4_H_7_O_4_N, H_2_L) is denoted as H_2_ (C_4_H_5_O_4_N). Thus, the “L” of aspartic acid is C_4_H_5_O_4_N, which is denoted as ASP. As such, various complex species in the solution are described as follows, where Kx are the critical stability constants:
[H(ASP)] = K1[H][ASP]LogK1 = 9.65[(H)_2_(ASP)] = K2[H(ASP)][H]LogK2 = 3.70[(H)_3_(ASP)] = K3[(H)_2_ASP][H]LogK3 = 1.90[Cu(ASP)] = K4[Cu][ ASP]LogK4 = 8.88[Cu(ASP)_2_] = K5[Cu][ ASP]^2^LogK5 = 15.6[Cu(H)(ASP)] = K6[Cu(ASP)][H]LogK6 = 3.70[Cu(H)(ASP)_2_] = K7[Cu(ASP)_2_][H]LogK7 = 4.00[(Cu)_2_(ASP)] = K8[Cu(ASP)][Cu]LogK8 = 1.51[(Cu)_2_(ASP)_2_] = K9[Cu(ASP)_2_] [Cu] LogK9 = 3.60[Cu(OH)] = K10[Cu][OH] LogK10 = 6.50[Cu(OH)_2_] = K11[Cu][OH]^2^LogK11 = 11.8[Cu(OH)_3_] = K12[Cu][OH]^3^LogK12 = 14.5[Cu(OH)_4_] = K13[Cu][OH]^4^LogK13 = 16.4[(Cu)_2_(OH)] = K14[Cu]^2^[OH] LogK14 = 8.20[(Cu)_2_(OH)_2_] = K15[Cu]^2^[OH]^2^LogK15 = 17.7[(Cu)_3_(OH)_4_] = K16[Cu]^3^[OH]^4^LogK16 = 35.2[Cu(NO_3_)] = K17[Cu][NO_3_] LogK17 = −0.13[Cu(NO_3_)_2_] = K18[Cu][NO_3_]^2^LogK18 = −0.60

Here, C_Cu_ is the sum of the Cu complexes and ions. Therefore:

C_Cu_ = [Cu] + K4[Cu][ASP] + K5[Cu][ ASP]^2^ + K6[Cu(ASP)][H] + K7[Cu(ASP)_2_][H] + 2K8[Cu(ASP)][Cu] + 2 K9[Cu(ASP)_2_] [Cu] + K10[Cu][OH] + K11[Cu][OH]^2^ + K12[Cu][OH]^3^ + K13[Cu][OH]^4^ + 2K14[Cu]^2^[OH] + 2K15[Cu]^2^[OH]^2^ + 3K16[Cu]^3^[OH]^4^ + K17[Cu][NO_3_] + K18[Cu][NO_3_]^2^

In the same manner, C_ASP_ (total amount of ASP species) and $C_{{\mathrm{NO}}_{3}^{-}}$ (total amount of ${\mathrm{NO}}_{3}^{-}$ species) can describes as follows:

C_ASP_ = [ASP] + K1[H][ASP] + K2[H(ASP)][H] + K3[(H)_2_(ASP)][H] + K4[Cu][ASP] + 2K5[Cu][ ASP]^2^ + K6[Cu(ASP)][H] + 2K7[Cu(ASP)_2_][H] + K8[Cu(ASP)][Cu] + 2 K9[Cu(ASP)_2_] [Cu]

$C_{{\mathrm{NO}}_{3}^{-}}$ = [NO_3_] + K17[Cu][NO_3_] + K18[Cu][NO_3_]^2^

From the simultaneous equations of C_Cu_ and C_ASP_ and $C_{{\mathrm{NO}}_{3}}$, the ratio of each complex can be calculated. 

### 2.2. Reduction Potential Measurements of Homogenized Cu Complexes

The reduction potentials of homogenized Cu complexes were measured via the cyclic voltammetry method (GAMRY, Reference 600, Potentiostat/Galvanostat/ZRA, called as CV). Platinum wire, Au plate, and Ag/AgCl electrodes were used as the counter electrode, working electrode, and reference electrode, respectively.

### 2.3. Preparation and Selenization Method of Cu-In Alloy Nanoparticles

Cu-In alloy nanoparticles were prepared as follows. Aqueous solutions of 8–10 mmol/L Cu(Cl)_2_•2H_2_O (Wako Chemical, >99%), 10 mmol/L In(Cl)_3_•4H_2_O (Wako Chemical, >97%), 0.5 mol/L aspartic acid (Wako Chemical, >97%), were introduced into a glass vessel and mixed well. As necessary, polyvinylpyrrolidone (Wako Chemical, PVP (K25) and/or PVP(K16–18)) was added. The pH was adjusted to 9 with a NaOH aqueous solution, and the final volume was adjusted to 40 mL. Then 10 mmol of a NaBH_4_ solution (10 mL, 30 °C) whose pH was similar to the metal complex solution was introduced into the glass vessel, and mixed at 30 °C for 1 h. The synthesized materials were collected, washed with EtOH, and dried. 

The synthesized Cu-In nanoparticles (1.5 g) were well dispersed into 7.5-mL 2-PrOH by ultrasonic wave irradiation for 60 min. The synthesized Cu-In alloy nanoparticle dispersion was spin-coated onto a Mo/glass substrate and dried at 200 °C. Selenization of the Cu-In nanoparticle film on a Mo/glass substrate was performed under a 9.6 × 10^−2^ Pa H_2_Se gas atmosphere (total pressure: 0.6 atm). The treatment temperature was changed from 300 °C to 575 °C at a constant treatment time of 1 h. 

### 2.4. Evaluation of the Synthesized Materials

The synthesized materials were evaluated by XRD (Rigaku Co., Ltd., Japan. MULTI FLEX, 40 kV, 20 mA), SEM (Hitachi Co., Ltd., Japan. SU8000), Raman spectroscopy (HORIBA JOBIN YVON, T64000). I-V measurements and quantum effectivity measurements were performed using cells formed by a CIS film (*p*-type semiconductor) and a CdS film (*n*-type semiconductor).

## 3. Results and Discussion

**Figure 1:**
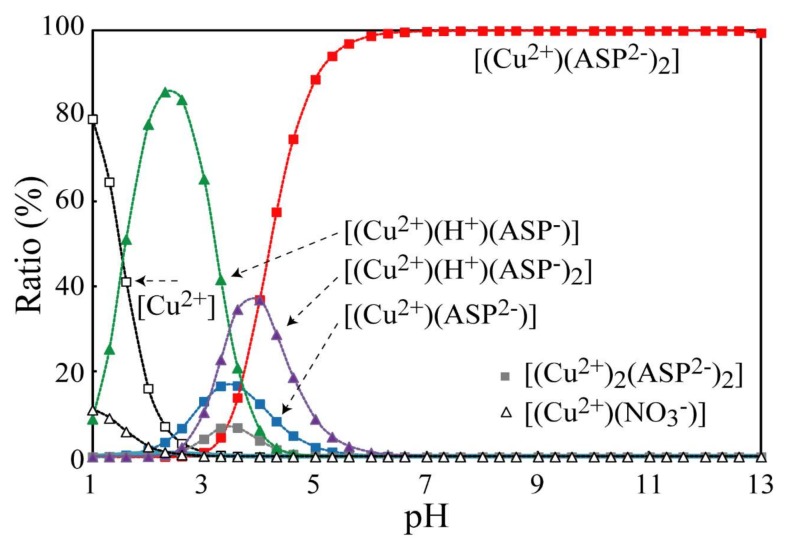
Results of the calculation in the cases of Cu-ASP-OH system as a function of pH, (C_Cu_ = 0.01 mol/L, $C_{{\mathrm{NO}}_{3}}$ = 0.02 mol/L, and C_ASP_ = 0.06 mol/L).

Figure 1 shows the calculation results for the Cu-aspartic acid-OH system (C_Cu_ = 0.01 mol/L, $C_{{\mathrm{NO}}_{3}}$ = 0.02 mol/L and C_aspartic acid_ = 0.06 mol/L), which reveal the relationship between the ratio of Cu complexes (vertical axis) and pH (horizontal axis). As already mentioned, aspartic acid (C_4_H_7_O_4_N, H_2_L) is denoted as H_2_ (C_4_H_5_O_4_N). Thus, the “L” of aspartic acid is C_4_H_5_O_4_N, which is denoted as ASP^2−^. Various Cu-aspartic acid complexes such as [(Cu^2+^)(H^+^)(ASP^−^)] and Cu ions form in acidic conditions, However, [(Cu^2+^)(ASP^2−^)_2_] is predominant in the pH region of 6–13. These results suggest that the Cu complex can be homogenized if the solution is adjusted to the above conditions. It should be noted that homogenization is successfully achieved in the case of C_ASP_/C_Cu_ > 6 (=0.06/0.01), which is not obtained by the addition of stoichiometric amounts. 

Table 1 summarizes the relationship between homogenized species and the solution condition calculated in the case of each complex reagent. The “L” of oxalic acid (H_2_L), EDA (L), NTA (H_3_L), EDTA(H_4_L), maleic acid (H_2_L), citric acid (H_3_L), and glycine (HL) are denoted as “oxa”, “EDA”, “NTA”, “EDTA”, “mal”, “cit”, and “gly”, respectively. The Cu complexes are restricted to [(Cu^2+^)(oxa^2−^)_2_] (pH 3–10), [(Cu^2+^)(gly^−^)_2_] (pH 6–12), [(Cu^2+^)(EDA)_2_] (pH 7–12) and [(Cu^2+^)(NTA^3−^)_2_] (pH 8–11). [(Cu^2+^)(EDTA^4−^)] (pH 5–10), [(Cu^2+^)_2_(OH^−^)_2_(mal^2−^)_2_] (pH 9–12), and [(Cu^2+^)_2_(OH^−^)_2_(cit^3−^)_2_] (pH 7–12) are predominant in the respective pH region. Similar to the case of aspartic acid, homogenization of Cu complex requires the addition of 1.5 times (NTA), two times (EDTA and EDA), three times (glycine), six times (maleic acid and citric acid), and 10 times (oxalic acid) of the complex reagents relative to Cu. On the other hand, the calculations for the In-complex reagents system demonstrate that In complexes can be restricted to [(In^3+^)(OH^−^)(EDTA^4−^)] (pH 10–12, C_EDTA_/C_In_ = 2) and/or [(In^3+^)(ASP^2−^)_2_] (pH 7–9, C_ASP_/C_In_ = 5), respectively.

Cyclic voltammetry measurements of these homogenized metal complexes show peaks at −0.87 (V (vs. Ag/AgCl)) (oxa), −0.94 (V (vs. Ag/AgCl)) (gly), −1.08 (V (vs. Ag/AgCl)) (EDA), −1.01 (V (vs. Ag/AgCl)) (ASP), −0.98 (V (vs. Ag/AgCl)) (NTA), −1.05 (V (vs. Ag/AgCl)) (EDTA), −1.00 (V (vs. Ag/AgCl)) (mal), and −1.03 (V (vs. Ag/AgCl)) (cit).

The XRD measurement results of the films electrodeposited at the corresponding voltage (1800 sec) show apparent peaks of metallic Cu (PDF#04-0836) along with peaks of the Au substrate (PDF#04-0784). Thus, these voltages indicate the reduction potential of homogenized metal complexes (Table 1). 

Here, the reduction potential of Cu metal from its oxide is clearly defined by the pH (i.e., potential-pH diagram). For example, that of Cu is −0.2 (V vs. SHE) [26]. On the other hand, that of Cu metal reduced from its complex shows another potential, which depends on the ligand species since the disconnection between Cu and the ligand is indispensable to reduce the Cu complex species (Table 1). The reduction potential shown in Table 1 can be recognized as that of Cu metal from its homogenized Cu complex, and it depends on the critical stability constants between Cu ion and ligands.

Among the consecutive stability constants and overall stability constants, the latter is relevant to the reduction of the Cu complex since the complex needs a perfect disconnection of the bonding between metal and ligands. The overall stability constants (K) can be described as follows:

(1) $${K=k}_{1}\cdot k_{2}{\cdot \cdot \cdot \cdot \cdot \cdot k}_{n}=\frac{{[\mathrm{ML}}_{n}]}{[M{\left(H_{2}O\right)}_{n}{][L]}^{2}}$$

**Figure 2:**
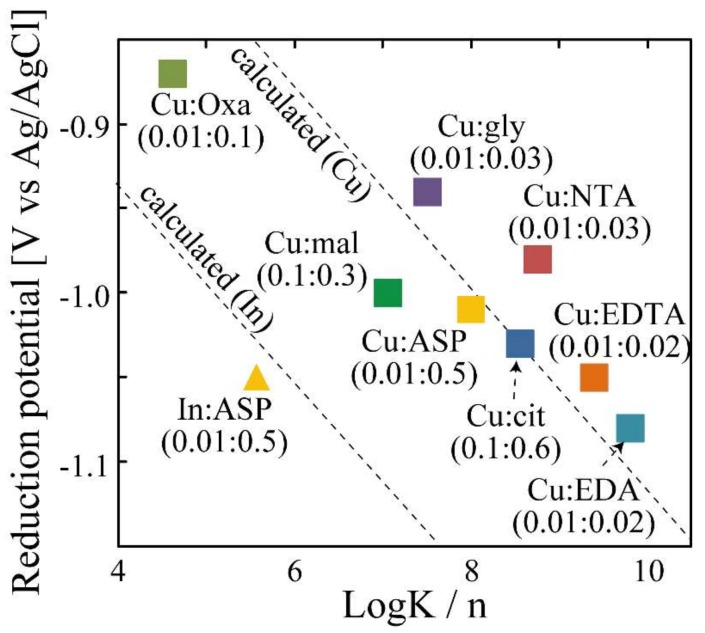
Relationship between LogK/n and reduction potential of homogenized Cu complexes. Reduction potential of Cu and In complexes calculated from Equations (8) and (9) were also inset in the figure (dotted line).

Figure 2 shows the relationship between LogK/*n* and the reduction potential of the homogenized Cu complexes. “n” means the valence of the metal in the complex. For example, n = 2 for [(Cu^2+^)(Ligand)] and n = 4 for [(Cu^2+^)_2_(Ligand)]. This relationship has a linear tendency. 

The reduction formula of [(M^n+^)(Ligand)] can be expressed as:

(2) $$\left[{(M}^{n+}\right){L]+\mathrm{ne}}^{-}\to M+L$$

By utilizing Nernst’s equation, the reduction potential of Equation (2) can be expressed as:

(3) $${E=E}_{c}^{0}-\frac{\mathrm{RT}}{\mathrm{nF}}\cdot \ln\frac{L}{\left[{(M}^{n+}\right)L]}$$

On the other hand, when the metal ion (M^n+^) and ligand (L) form a complex with the stability constants K, K is expressed as:

(4) $$M^{n+}+L\to \left[{(M}^{n+}\right)L]$$

(5) $$K=\frac{\left[{(M}^{n+}\right)L]}{\left[M^{n+}\right][L]}$$

Thus, the standard electrode potential of metal complex can be summarized as:

(6) $${E=E}_{c}^{0}+\frac{\mathrm{RT}}{\mathrm{nF}}\cdot \mathrm{lnK}+\frac{\mathrm{RT}}{\mathrm{nF}}\to \ln[M^{n+}]$$

(7) $$E_{c}^{0}{=E}_{0}-\frac{\mathrm{RT}}{\mathrm{nF}}\cdot \mathrm{lnK}$$

Here, the temperature (T) is 298 (K), gas constant (R) is 8.31 (J K^−1^ mol^−1^), the Faraday constant (F) is 96,500 (C mol^−1^), and the reduction potential of “bare” Cu ion (E_0_) is −0.525 (V vs. Ag/AgCl) [27]. Therefore, the reduction potential of Cu metal complex can be expressed as:

(8) $$E_{c}^{0}=-0.525-0.059\cdot \frac{\mathrm{logK}}{n}$$

In the same manner, considering the reduction potential of “bare” In ion (E_0_ = −0.700 (V vs. Ag/AgCl) [28]), the reduction potential of the In metal complex can be expressed as:

(9) $$E_{c}^{0}=-0.700-0.059\cdot \frac{\mathrm{logK}}{n}$$

**Figure 3:**
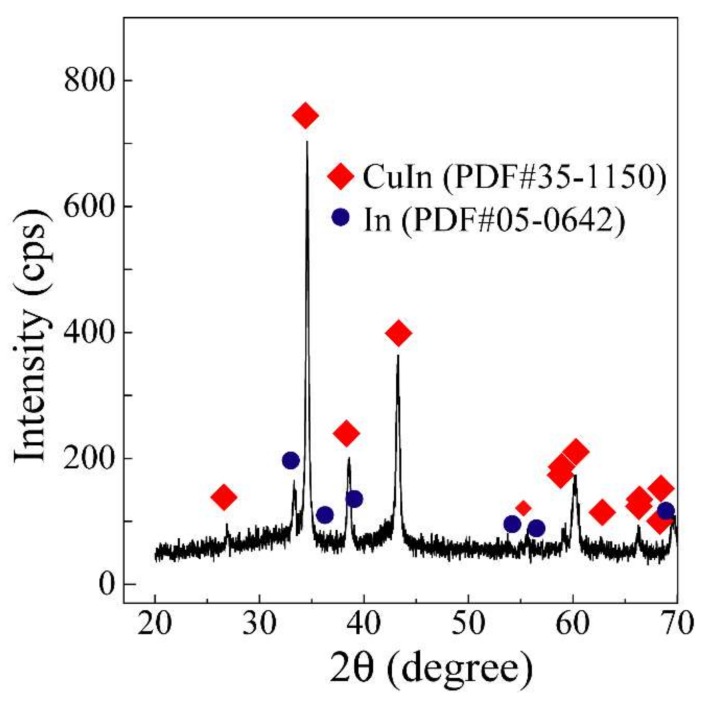
XRD profile of the material synthesized from the 0.01 M CuCl_2_-0.01 M InCl_3_-0.50 M ASP aqueous solution (pH 9, 30 °C, reaction time was 1 h).

The relationship between the reduction potential and the stability constants of Cu and In complexes calculated from Equations (8) and (9) (dotted line in Figure 2) are consistent with the measured values. Therefore, the reduction potential of the homogenized metal complex can be determined from these equations and the overall stability constants. In this study, alloy nanoparticles are synthesized using reducing agents with a specific potential. Thus, the low potential deference of both metal complexes, ∆(Cu-In), is significant in the synthesis of alloy nanoparticles. Taking these facts into consideration, it is expected that aspartic acid is a suitable ligand for Cu and In since ∆(Cu-In) is low (0.04). Actually, the XRD peaks (Figure 3) according with CuIn alloy (PDF#35-1150), Cu_2_In alloy (PDF#42-1475), and In (PDF#05-0642) are detected from the film electrodeposited by using 0.01 M Cu(NO_3_)_2_-0.01 M In(NO_3_)_3_-0.50 M aspartic acid aqueous solution (pH 9) at −1.06 V (V (vs. Ag/AgCl)). These results indicate that Cu-In alloy nanoparticles can be synthesized under this condition. 

Thus, chemical reduction was performed by using 0.01 M Cu(NO_3_)_2_-0.01 M In(NO_3_)_3_-0.50 M aspartic acid aqueous solution (pH 9) and 0.1 M NaBH_4_ solution as a reducing reagent. Due to the coordination of water molecules to In in the presence of ${\mathrm{NO}}_{3}^{-}$ ion [27], the synthesized precipitate was re-dissolved into solution during filtration and the drying process. Therefore, metal salts were changed from nitrates to chlorides. Cu-chloride or In-chloride complexes are not formed in this solution condition.

From the XRD profile of the material synthesized from the 0.01 M CuCl_2_-0.01 M InCl_3_-0.50 M aspartic acid aqueous solution (pH 9, 30 °C, reaction time of 1 h) and 0.1 M NaBH_4_ solution, many peaks corresponding to CuIn (PDF#35-1150) and small peaks of In (PDF#05-0642) are clearly observed. The recovery rate is >98%. It is well known that In can co-exist in the η phase (Cu_2_In) below 157 °C, as shown in the Cu-In binary phase diagram [28,29]. It is also true that the CuIn alloy phase is also obtained frequently in thermodynamically slow syntheses, leading to a nonequivalent state. This experiment used thermodynamically slow conditions (e.g., 30 °C) [30,31,32,33,34]. For example, a mixture of CuIn/In/Cu_2_In has been synthesized in other reports [21,35].

From the above results, it is clear that Cu-In alloy nanoparticles can be synthesized in the aqueous phase at room temperature. However, their particle size varies widely (20–600 nm), which is not suitable for CIS-based printable solar batteries. Therefore, we tried to control the particle diameter (PD) and standard deviation (SD) of the synthesized alloy nanoparticles.

**Figure 4:**
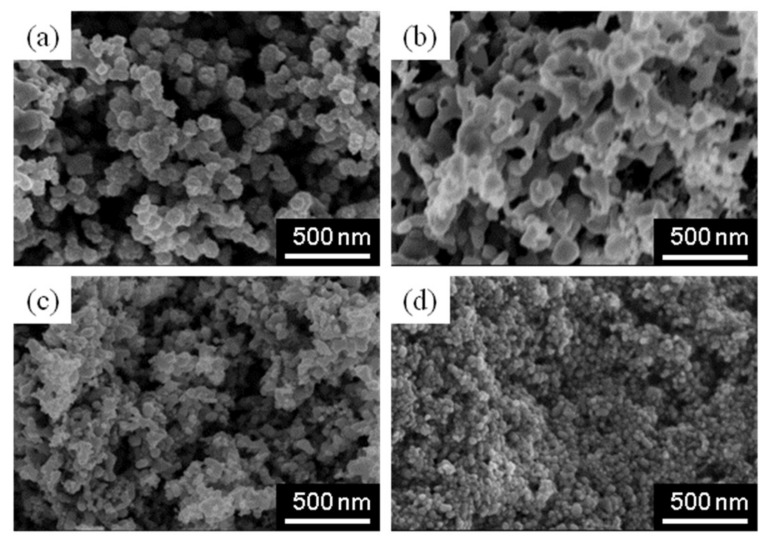
SEM micrographs of Cu-In alloy nanoparticles synthesized with (**a**) 2.5 mM CTAB; (**b**) 2.5 mM SDS; (**c**) 2.5 mg/50 mL PVP(K25); and (**d**) 2.5 mg /50 mL PVP(K16–18), respectively.

Figure 4 shows the SEM micrographs of Cu-In alloy nanoparticles synthesized with (a) 2.5 mM CTAB, (b) 2.5 mM SDS, (c) 2.5 mg/50mL PVP(K25), and (d) 2.5 mg/50 mL PVP(K16–18), respectively. The PD/SD calculated from 200 particles drastically changes (i.e., 88 nm/41 (CTAB 2.5 mM), 76 nm/31 (SDS, 2.5 mM), 31 nm/12 (PVP(K16–18), 2.5 mg/50 mL), 47 nm/19 (PVP(K25), and 2.5 mg/50 mL)). Here, the basic structures of PVP(K16–18) and PVP(K25) are almost the same, but the polymerization degree of the vinyl groups differ. The length of the former is ca. 79 nm, while that of latter is ca. 350 nm. On the other hand, the synthesized particle size is ca. 50 nm.

Taking the previously report, which mentioned the relationship between the length of the vinyl groups in PVP and the secondary particle size, into consideration [36], the particle size control mechanism is as follows: In the case of PVP(K16–18), Cu-In nanoparticles do not aggregate since the diameter and the length of PVP are almost the same. On the other hand, many Cu-In nanoparticles form secondary particles when PVP(K25) is used as the size-control reagent due to the intertwining of long functional groups around the particles.

**Figure 5:**
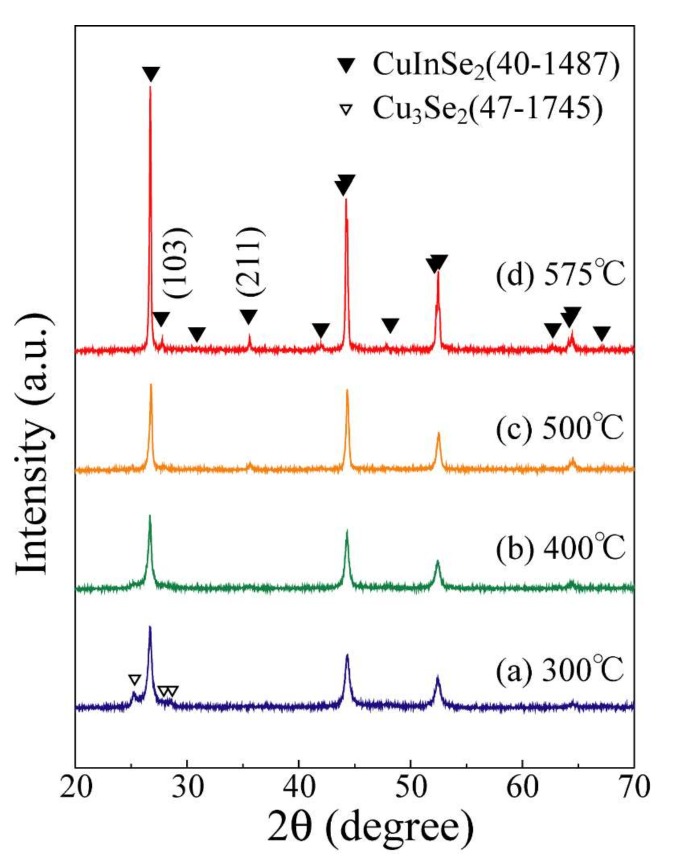
XRD profiles of Cu-In precursor films selenized at 300, 400, 500, and 575 °C.

Cu-In precursor films with relatively flat surfaces are successfully spin-coated onto the substrate via a 2-propanol dispersion of Cu-In alloy nanoparticles synthesized with PVP(K16–18). Figure 5 shows the XRD profiles of the Cu-In precursor films after selenization at 300, 400, 500, and 575 °C. The peaks of the CuIn alloy phase (PDF#35-1150) disappear, while those the CuInSe_2_ phase (PDF#40-1487) and the Cu_3_Se_2_ phase (PDF#47-1745) appear when Cu-In precursor films are selenized at 300 °C. As the treatment temperature increases to 400 °C, only the peaks of the CuInSe_2_ phase (PDF#40-1487) are detected. However, at a treatment temperature of 575 °C, the peaks of the (103) and (211) planes of the CuInSe_2_ phase, which indicate the existence of the chalcopyrite structure [37], are detected. Since the chalcopyrite structure of CuInSe_2_ phase is essential to construct effective solar cells [38], the selenide temperature used in this experiment was 575 °C. Here, the Cu_2_Se phase is sometimes co-synthesized with the CuInSe_2_ phase as a byproduct [39]. However, we cannot identify the CuInSe_2_ and Cu_2_Se from the XRD profile because both have the same primitive lattice and almost the same lattice constants [40,41,42,43]. 

**Figure 6:**
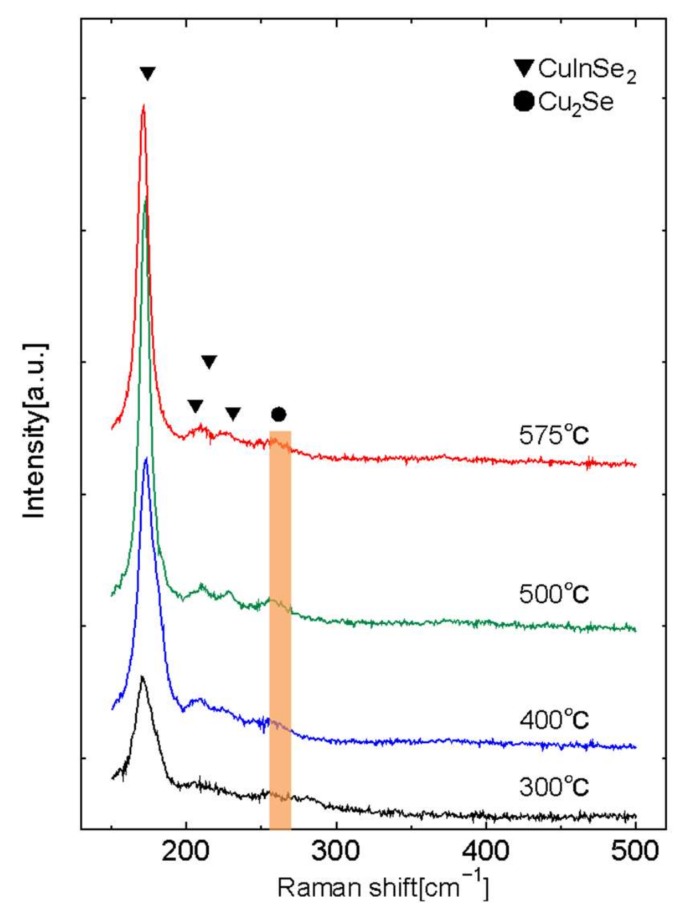
Results of Raman scattering measurements of the CIS films selenized at 300, 400, 500, and 575 °C.

The existence of Cu_2_Se was evaluated by the Raman scattering measurement method. Figure 6 shows CIS films selenized at 300, 400, 500, and 575 °C. The peaks of CulnSe_2_ are detected at 174 cm^−1^ (A_1_ vibrational mode), 206 cm^−1^ (E mode), 215 cm^−1^ (B_2_,E mode) and 231 cm^−1^ (B_2_,E mode) [44], while a weak peak of Cu_2_Se is also detected at 262 cm^−1^ [45]. Thus, the main phase of the CIS films synthesized in this study is CulnSe_2_. Nevertheless, a very small amount of Cu_2_Se phase is involved. Taking the pseudo-binary phase diagram of Cu_2_Se-In_2_Se_3_ into consideration [46], it expected that Cu_2_Se will separate under the conditions of In_2_Se_3_ < 50 mo1% and T < 500 °C since CulnSe_2_ (α phase) does not have a solid solution region. To restrict the synthesis of byproducts, the ratio of Cu against In should be less than 1. 

**Figure 7:**
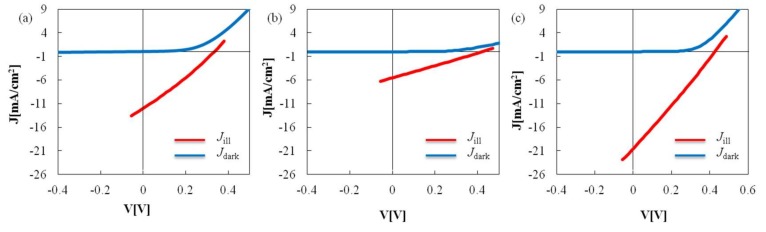
J-V measurement results of CIS films which constituted by using Cu-In nanoparticles synthesized at (**a**) PVP(K16–18) and Cu/In = 0.91; (**b**) PVP(K25) and Cu/In = 0.91; and (**c**) PVP(K25) and Cu/In = 0.81, under AM 1.5G illumination and in the dark.

Figure 7 shows the J-V measurement results of the CIS films, which were prepared using Cu-In nanoparticles synthesized at (a) PVP(K16–18) and Cu/In = 0.91; (b) PVP(K25) and Cu/In = 0.91; and (c) PVP(K25) and Cu/In = 0.81. The measurements were performed under irradiation of AM 1.5-G illumination and under dark conditions. Table 2 summarizes the open circuit potential (V_OC_ [mV]), short circuit current density (J_SC_ [mA/cm^2^]), fill factor (FF), and conversion efficiency (η[%]) of each CIS solar cell. 

The CIS films synthesized from Cu-In alloy nanoparticles formed in an aqueous solution at room temperature act as solar cells. The maximum conversion efficiency (η) is 2.30%. These results indicate that the aqueous phase synthesized Cu-In alloy nanoparticles can applied to CIS-based printable solar batteries, although the efficiency of the CIS solar cell synthesized in this experiment is lower than that of the CIS solar cell synthesized under the gas phase condition (for example, η = 20.9% [14]).

There are three reasons why the efficiency of the solar battery synthesized in this experiment is lower than that synthesized in the gas phase: (1) recombination loss, (2) parasitic loss; and (3) optical loss. These losses induce the loss of (1) Voc, (2) FF, Jsc, Voc, and (3) Jsc [17]. The series resistance (Rse), parallel conductance (Gsh), ideality factor (n), saturation current density (J_0_) of the solar battery’s equivalent circuit can be calculated as follows: The J-V properties of the solar battery’s equivalent circuit under darkness condition is expressed as [47]:

(10) $${J=J}_{0}\exp\left[\frac{q}{\mathrm{nkT}}\left(V-\mathrm{RseJ}\right)\right]+\mathrm{GshV}$$

Here, J_0_ is the saturation current density (mA/cm^2^), q is the quantum of electricity (1.602 × 10^−19^ (C)), and n is ideality factor of the diode. k is Boltzmann’s factor (1.380 × 10^−23^ (J/K)), and T is temperature (K). Rse is the series resistance (Ω cm^2^) and Gsh is the parallel conductance (mS/cm^2^). If Equation (10) is differentiated with respect to V, Equation (11) is obtained:

(11) $$\frac{\mathrm{dJ}}{\mathrm{dV}}{=J}_{0}\left[\frac{q}{\mathrm{nkT}}\left(V-\mathrm{RseJ}\right)\right]\exp\left[\frac{q}{\mathrm{nkT}}V\right]+\mathrm{Gsh}$$

In the region V < 0, $J_{0}\left[\frac{q}{\mathrm{nkT}}\left(V-\mathrm{RseJ}\right)\right]\exp\left[\frac{q}{\mathrm{nkT}}V\right]$ approaches to 0 from the plot of V against to dJ/dV. Thus, the value where dJ/dV becomes constant is equivalent to Gsh in the region V < 0. 

On the other hand, if Equation (10) is differentiated with respect to J, it expressed as:

(12) $$\frac{\mathrm{dV}}{\mathrm{dJ}}=\mathrm{Rse}+\frac{\mathrm{nkT}}{q}J^{-1}$$

Thus, the y-intercept and gradient of the plot of J^−1^ against to dV/dJ are equal to Rse and nkT/q, respectively. From these, the value of Gsh, Rse, and n become clear. Transformation of Equation (10) yields, Equation (13):

(13) $$\ln\left(J-\mathrm{GshV}\right)=\frac{q}{\mathrm{nkT}}\left(V-\mathrm{RseJ}\right){+\mathrm{lnJ}}_{0}$$

Thus, the y-intercept of the plot of V − RseJ against to ln(J − GshV) is equal to lnJ_0_. From the relationship of these equations, Rse, Gsh, n, and J_0_ can be calculated. 

**Figure 8:**
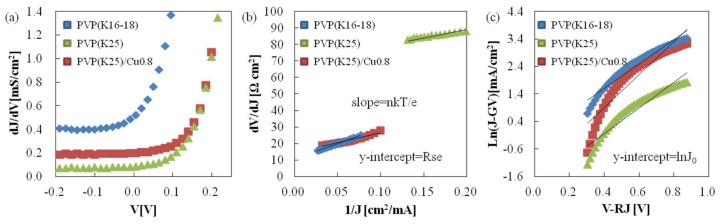
(**a**) dJ/dV vs. V curves for Gsh evaluation; (**b**) dV/dJ vs. 1/J plot for the determination of Rse and n; and (**c**) semilogarithmic plot of (j-GshV/A) vs. V-RJ for determination of J_0_.

Figure 8 shows the (a) dJ/dV vs. V curves to evaluate Gsh; (b) dV/dJ vs. 1/J plot to determine Rse and n; and (c) semi-logarithmic plot of (j-GshV/A) vs. V-RJ to determine J_0_. Table 3 summarizes the Gsh, Rse, n, and J_0_, of the CIS devices synthesized in this study, as well as those for the gas phase condition. n and J_0_ of the CIS devices synthesized in this experiment are higher than those of the gas phase synthesized CIS devices. These results mean that the CIS devices synthesized in this experiment involve many recombination losses, which lowers the open circuit potential.

**Figure 9:**
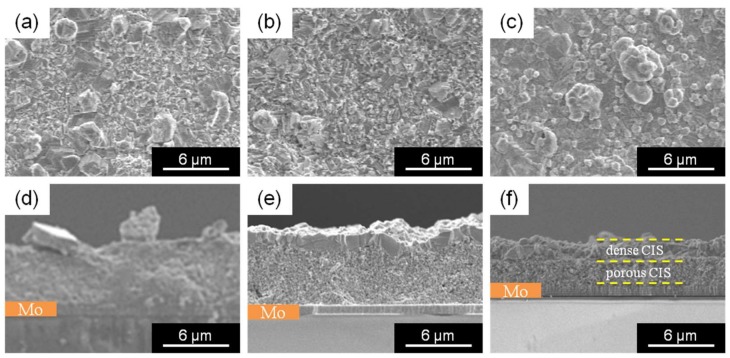
SEM images (surface morphology and cross-section) of the CIS solar cell based on Cu-In nanoparticles using (**a**,**d**) PVP(K16-18) and Cu/In = 0.91, (**b**,**e**) PVP(K25) and Cu/In = 0.9, (**c**,**f**) PVP(K25) and Cu/In = 0.81.

Figure 9 shows the SEM images (surface morphology and cross-section) of the CIS solar cells based on Cu-In nanoparticles using (a,d) PVP(K16–18) and Cu/In = 0.91, (b,e) PVP(K25) and Cu/In = 0.9, and (c,f) PVP(K25) and Cu/In = 0.81. In each case, it is apparent that the cell has a rough surface, which induces the lack of p-n junctions [19]. Moreover, crystal growth is limited to the surface area of the CIS films (<1 μm). The crystallinity inside the CIS films is low, and the thickness of the CIS solar cell is 4–6 μm, which is two or three times thicker than that of the effective CIS solar battery [48]. These characteristics increase the series resistance, leading to recombination loss that eventually results in the loss of FF, Jsc, and Voc. These results mean that the smoothing of the CIS films and improving the crystallinity and thickness induce the solar cells properties.

## 4. Conclusions

The calculations and CV measurements of homogenized complex species indicate that aspartic acid is a suitable ligand for Cu and In because Δ(Cu-In) is low (0.04). Under the appropriate conditions (0.01 M CuCl_2_-0.01 M InCl_3_-0.50 M aspartic acid aqueous solution (pH 9, 30 °C, reaction time was 1 h) and a 0.1 M NaBH_4_ solution), CuIn alloy nanoparticles with In small peaks are successfully synthesized with a recovery rate of >98%. CIS films synthesized from Cu-In alloy nanoparticles act as solar cells with the maximum conversion efficiency of 2.30%. The results indicate that aqueous phase synthesized Cu-In alloy nanoparticles can be applied to CIS-based printable solar battery. This method should be applicable to various alloy synthesis systems. 

## Figures and Tables

**Table 1:** Relationship between homogenized species and solution condition calculated, and reduction potential in the case of oxalic acid, glycine, ethylenediamine (EDA), nitrilotriacetic acid (NTA), etylenediaminetetraacetic acid (EDTA), malic acid, and citric acid.

Complex Reagent	Concentration of Cu (mol/L)	Concentration of Complex Reagent (mol/L)	Homogenized Species	Ratio of Homogenized Species (%)	Reduction Potential [V(vs. Ag/AgCl)]
oxalic acid	0.01	0.1	[(Cu^2+^)(oxa^2−^)_2_]	100	−0.87
glycine	0.01	0.03	[(Cu^2+^)(gly^−^)_2_]	100	−0.94
EDA	0.01	0.02	[(Cu^2+^)(EDA)_2_]	100	−1.08
ASP	0.01	0.06	[(Cu^2+^)(ASP^2−^)_2_]	100	−1.01
NTA	0.01	0.03	[(Cu^2+^)(NTA^3−^)_2_]	100	−0.98
EDTA	0.01	0.02	[(Cu^2+^)(EDTA^4−^)]	96.2	−1.05
malic acid	0.01	0.03	[(Cu^2+^)_2_(OH^−^)_2_(mal^2−^)_2_]	92.8	−1.00
citric acid	0.01	0.06	[(Cu^2+^)_2_(OH^−^)_2_(cit^3−^)_2_]	93.8	−1.03

**Table 2:** PV parameters evaluated from the J-V curves for the CIS solar cells.

Sample	V_OC_ (mV)	J_SC_ (mA/cm^2^)	FF	η (%)
PVP(K16–18), Cu/In = 0.91	330	12.0	0.28	1.12
PVP(K25) Cu/In = 0.91	420	5.53	0.26	0.60
PVP(K25) Cu/In = 0.81	430	20.5	0.26	2.30

**Table 3:** Shunt conductance (Gsh), series resistance (Rse), ideality factor (n), and saturation current density (J_0_) of CIS devices synthesized in this study and also gas phase condition.

Sample	Gsh (mS/cm^2^)	Rse (Ω cm^2^)	n	J_0_ (mA/cm^2^)
PVP(K16–18)	0.40	10.8	7.06	0.86
PVP(K25)	0.08	68.4	4.00	0.15
PVP(K25)/Cu0.8	0.20	14.3	4.71	0.23
CIGS(gas phase) (η = 15.5%)	0.05	0.20	1.50	2 × 10^−6^
